# Having a chat and then watching a movie: how social interaction synchronises our brains during co-watching

**DOI:** 10.1093/oons/kvae006

**Published:** 2024-03-25

**Authors:** S De Felice, U Hakim, N Gunasekara, P Pinti, I Tachtsidis, A Hamilton

**Affiliations:** Department of Psychology, University of Cambridge, 2 Free School Lane, CB2 3RF, UK; Institute of Cognitive Neuroscience, University College London, Alexandra House, 17-19 Queen Square, London WC1N 3AZ, UK; Department of Medical Physics and Biomedical Engineering, University College London, Malet Place Engineering, Gower St, London WC1E 6BT, UK; Department of Medical Physics and Biomedical Engineering, University College London, Malet Place Engineering, Gower St, London WC1E 6BT, UK; Department of Medical Physics and Biomedical Engineering, University College London, Malet Place Engineering, Gower St, London WC1E 6BT, UK; Centre for Brain and Cognitive Development, Birkbeck College, University of London, 33 Torrington place, London WC1E 7JL, UK; Department of Medical Physics and Biomedical Engineering, University College London, Malet Place Engineering, Gower St, London WC1E 6BT, UK; Institute of Cognitive Neuroscience, University College London, Alexandra House, 17-19 Queen Square, London WC1N 3AZ, UK

**Keywords:** Brain-to-brain synchrony, social interaction, cowatching, wavelet coherence, fNIRS hyperscanning

## Abstract

How does co-presence change our neural experience of the world? Can a conversation change how we synchronise with our partner during later events? Using fNIRS hyperscanning, we measured brain activity from 27 pairs of familiar adults simultaneously over frontal, temporal and parietal regions bilaterally, as they co-watched two different episodes of a short cartoon. In-between the two episodes, each pair engaged in a face-to-face conversation on topics *un*related to the cartoon episodes. Brain synchrony was calculated using wavelet transform coherence and computed separately for real pairs and shuffled pseudo) pairs. Findings reveal that real pairs showed *increased* brain synchrony over right Dorso-Lateral Pre-Frontal cortex (DLPFC) and right Superior Parietal Lobe (SPL), compared to pseudo pairs (who had never seen each other and watched the same movie at different times; uncorrected for multiple comparisons). In addition, co-watching after a conversation was associated with greater synchrony over right TPJ compared to co-watching before a conversation, and this effect was significantly higher in real pairs (who engaged in conversation *with each other*) compared to pseudo pairs (who had a conversation with someone else; uncorrected for multiple comparisons). The present study has shed the light on the role of social interaction in modulating brain synchrony across people not just during social interaction, but even for *subsequent non-social* activities. These results have implications in the growing domain of naturalistic neuroimaging and interactive neuroscience.

## BACKGROUND

Watching a movie might be considered as a rather ‘solo’ activity, which does not need companionship. However, even though this may seem unnecessary, people like to watch movies together. Why? Several studies show brain entrainment (inter-subject correlation in brain response) during solo movie watching, but does that change in joint watching?

The phenomenon by which attending to the same movie stimuli elicits similar neural activity across brains has been referred to as *inter-subject correlation* (ISC, (Hasson & Frith, 2016). Using fMRI, (Hasson et al., 2004) found that five different individuals watching a movie showed similar neural response in occipital, parietal and temporal areas. Between-brain synchronisation was found to extend beyond typical auditory and visual sensory-processing cortices to high-level association areas. This made the authors conclude that ISC reflected shared understanding of the movie narrative. Following this work, a number of studies confirmed this interpretation, showing ISC over areas involved in reasoning and abstract thinking, including pre-frontal and frontal regions (Jääskeläinen et al., 2008), and extending from visual stimuli to speech comprehension (Wilson et al., 2008), and from fMRI to EEG group-analysis (Poulsen et al., 2017).

Furthermore, similar neural representations have been found during interpretation and recall of shared events. For example, when people were given different interpretations of an ambiguous story, ISC was greater between individuals who were given the same interpretation (Yeshurun et al., 2017), and the same results replicated when participants were free to develop their own interpretation of a movie showing interacting abstract shapes (Nguyen et al., 2019). Likewise, in an fMRI study, (Chen et al., 2017) found that neural patterns were more similar between people recalling the same event than between recall and perception of that event.

Recently, (Madsen & Parra, 2022) cleverly demonstrated that ISC is the result of effective cognitive processing: they presented participants with informative videos in an attentive and distracted condition, while measuring their neural activity (via EEG) and physiology including heart rate and breathing, as well as gaze position, pupil size and head movement. ISC was linked to attentional state and predicted subsequent recall of information presented in the videos. These findings support the notion that ISC is a good biomarker of how similarly different brains process the world around them.

If ISC reflects shared understanding, how is it modulated by relational dynamics? In other words, would ISC differ between people that tend to be psychologically closer and ‘understand each other better’, like partners, family and friends? Using fMRI, (Parkinson et al., 2018) collected information about the social network proximity between undergraduate students, and used it to predict similarity in neural response during movie (solo) watching. They found that ISC increased as distance in real-life social network decreased.

**Figure 1:**
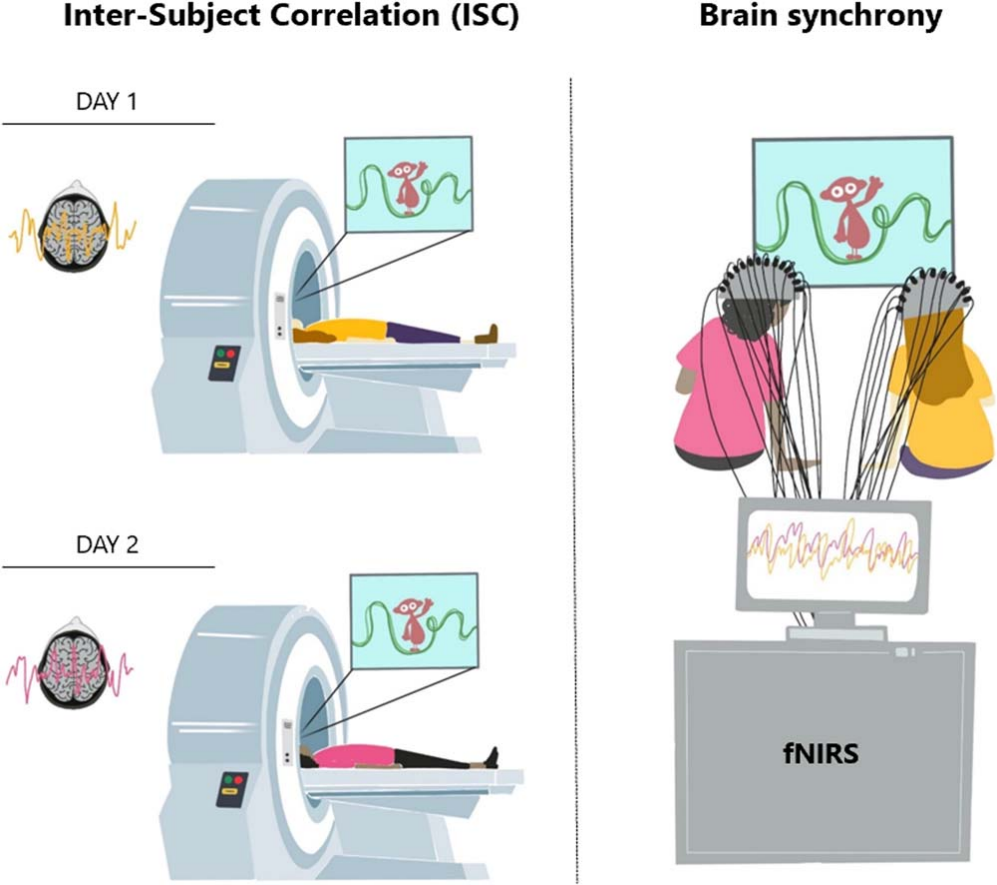
**Inter-subject correlation vs Brain synchrony.** Left: on day 1, one participant is watching a cartoon while receiving a brain scan. On day 2, another participant is undergoing the same procedure, watching the same cartoon and receiving a brain scan. The neural responses is than compared across different participants who did the same task at separate times and on their own. The similarity across neural responses to the same stimulus (e.g. the cartoon) is computed as inter-subject correlation. Right: On the same day, two participants watch a cartoon together, in real time, next to each other. Their brains response is recorded simultaneously, and the coherence between the two signals is computed as brain-to-brain synchrony.

The work cited so far specifically looked at how ‘aligned’ different individual brains were in response to specific contexts, as people were attending to different stimuli *alone*. However, they cannot answer questions about real-time interactive minds. Going beyond single-brain scanning, hyperscanning studies look at *brain-to-brain synchrony* by recording brain activity from multiple people simultaneously (see Fig. 1), and therefore can give information about the real-time neural dynamics between interactive brains (Babiloni & Astolfi, 2014).

In the context of movie-watching, Azhari and colleagues designed a series of hyperscanning studies where they measured brain-to-brain synchrony in parent–child dyads (Azhari et al., 2019, 2020, 2021, 2023). In their paradigm, the child sits on their parent’s lap to co-watch a series of short cartoons, while brain activity of the parent and the child is recorded simultaneously via fNIRS. Findings showed that real father-child dyads exhibited greater synchrony than pseudo dyads (i.e. shuffled parent–child pairs) in the medial left pre-frontal cortex (mPFC). Also, this was modulated by father’s age [1], parenting stress (Azhari et al., 2019) and maternal anxiety (Azhari et al., 2023), with older, more stressed and more anxious parents resulting in less synchrony. Interestingly, co-parenting couples attending to social salient signals (e.g. a child laughing) also exhibited greater synchrony when they were physically in the same room, compared to attending to the same stimuli at separate times, and significantly more than fake couples [3].

Taken together, results from studies of brain-to-brain dynamics suggest that not only do different brains respond similarly to the same reality, but that such similarity is modulated by relationship closeness, possibly reflecting affinity in the way people perceive, experience and make sense of the world. However, some questions still need to be addressed. All the studies discussed so far considered long-term social dynamics, i.e. relationship that built over several years (parent–child, romantic couples, and friends) and did not consider real-time (short-term) social interaction. It remains unclear whether face-to-face communication (e.g. having a conversation) modulates synchrony in brain activity between people as they co-experience the world around them.

Several studies have looked at how brain synchrony varies during – and as a function of – social interaction [8, 13, 15, 19, 24, 28]; for some reviews on this see [12, 25]. For example, Pan and colleagues found higher inter-brain synchrony across three participants as they analysed an ancient Chinese poem together (cooperative condition) versus solo-sessions (independent condition). In other study, [Nguyen et al., [16]] showed that inter-brain synchrony was predicted by turn-taking events during natural conversations between a child and their mother. This literature demonstrates that brain synchrony reflects inter-personal dynamics arising *during* social interaction. However, less is known about whether and how social interaction modulates brain synchrony during *later non-interactive* events. In other words, how does having a conversation aligns brain activity of people afterwards, as they engage in later (non-interactive) experiences, e.g. watching a movie?

An attempt to answer the question of whether having previously engaged in social interaction modulates ISC during later events is an fMRI study by (Sievers et al., 2020). In their paradigm, participants’ brains were first scanned individually during presentation of novel movie clips with ambiguous narratives. Then participants were assembled into small groups and asked to reach a consensus (via conversation) about each movie clip’s narrative. Finally, participants received a second brain scan, during which they were presented with the same clips as well as new ones from the same movies. Results revealed more ISC after conversation, and distinctive patterns of similarity in brain activity were observed within members of the same group, a finding interpreted by the authors as reflecting the group’s unique discussion. Sievers and colleagues’ work demonstrated the effect of real-world conversation in modulating ISC to later stimuli. However, by comparing neural response across solo brains, this work cannot inform our understanding of how people’s brains *synchronise* as they *co-*experience the world, nor can it show how a conversation changes inter-brain synchrony during shared experiences.

The distinction between *ISC* and *brain-to-brain synchrony* is relevant as it has implications for building cognitive models of how people process reality and interact with others. While neural ISC gives information on how similarly people’s brain respond to a given context, hyperscanning studies can capture brain-to-brain synchrony and describe complex dynamics *between interactive brains*, as they continuously and mutually adapt over their interaction. In addition, the question remains as to whether the ISC observed by Sievers and colleagues specifically reflects shared common-ground over a given topic (i.e. building a consensus about ambiguous narratives) or could potentially arise from any social interaction episode. The contribution of social interaction alone is difficult to disentangle in Sievers’s study, as while increased ISC after conversation extended to novel clips, these were still part of the same movie, and thus were related to the conversation content.

The question we ask here is not whether similarity in brain responses reflects shared understanding of a given experience (as this has been convincingly demonstrated elsewhere, e.g. see Hasson & Frith, 2016 for a review). Rather, we ask what is the minimal social factor that is able to modulate later brain synchrony between people: in other words, is talking *about* the experience necessary to observe conversation-related increase in brain coherence, or is social interaction per se between two people (e.g. having a conversation *unrelated* to the experience) enough to further attune their brain responses for later events?

In this study, we invited pairs of young adults to watch an episode of the BBC cartoon DipDap, as they sit next to each-other, while we measure their brain activity using fNIRS (Fig. 1). DipDap is a children’s cartoon with no verbal content, each episode lasting 2 minutes, that shows the adventures of an animated puppet (DipDap), who faces a series of unexpected challenges drawn on the screen by a line. After the first co-watching phase, participants engaged in a 20-minute conversation on unrelated topics (prompts for the conversation were provided, including sharing facts about exotic animals and musical instruments). They then took part in a second co-watching phase, when they were presented with a new episode of the same cartoon.

Note that all participants were familiar pairs (e.g. friends, flatmates or partners). This was dictated by the fact that data was collected during the covid-19 global pandemic, where UK government only allowed people within the same household to meet and interact face-to-face. We acknowledge this is a major limitations of this study and future work should use similar paradigm to compare familiar versus stranger dyads.

We analyse our data by using wavelet coherence transform analysis (Grinsted et al., 2004) to quantify the degree of similarity in brain signals between people as they watch the movie together. For this reason and consistently with previous work using similar method (e.g. Cui et al. 2012), we use the term ‘brain coherence’ as a synonym of brain synchrony. By comparing brain coherence computed from real-dyads to brain coherence computed from pseudo-dyads, separately for co-watching pre-conversation and co-watching post-conversation, we ask two questions: 1) is brain coherence during co-watching greater between real dyads (who sit next to each other and are familiar with each other) different from pseudo-dyads (i.e. baseline for stimulus-related activity)? And 2) what is the effect of an *unrelated* conversation on brain coherence during later co-watching?

## MATERIALS AND METHODS

### Participants

62 volunteers took part in the study, paired in 31 dyads. Participants were recruited via online platforms including university participant databases and social media, as well as flyers placed at local libraries and cafes around university campus. 1 dyad was excluded from the final sample due to data recording failure, and 3 dyads did not pass the pre-processing data quality check (see nirs signal processing section). The final sample included 27 dyads (N = 54, 34 females, 1 non-binary, age range = 19-37, age mean (sd) = 26.61 (4.76), years of education mean (sd) = 19.66 (2.99)). Demographic information are reported in Table 1. All participants gave written consent to participate in the study and were reminded of their right to withdraw at any point.

Data collection took place during a time of severe covid-19 restrictions, which made it necessary for both participants in each pair to be from the same household in order for them to participate in a face to face experiments (with no mask on). In our study, there were 17 ‘friends’ dyads, 10 ‘romantic’ dyads and 3 ‘flatmate’ dyads. The average relationship duration was 6.95 years (sd = 4.45). Overall, on a scale from 1 (not at all) to 5 (very), they reported to be close to their partner on average 4.43 (sd = 0.77), with no significant difference across dyad sub-groups.

**Table 1:** | MNI coordinates for the 8 ROIs included in the analysis

**Region**	**Laterality**	**X**	**Y**	**Z**
DLPF	R	44	34	28
DLPF	L	−46	30	30
vPM	R	64	−4	20
vPM	L	−58	−8	28
TPJ	R	58	−56	18
TPJ	L	−54	−56	22
SPL	R	37	−63	59
SPL	L	−40	−64	53

MNI coordinates for the centre of each ROIs as taken from neurosynth database (https://neurosynth.org/). DLPF = Dorso-Lateral Pre-Frontal cortex; vPM = ventral Pre-Motor cortex; TPJ = Temporo-Parietal Junction; SPL = Superior Parietal Lobe; L = Left; R = Right.

### Materials and procedure

**Figure 2:**
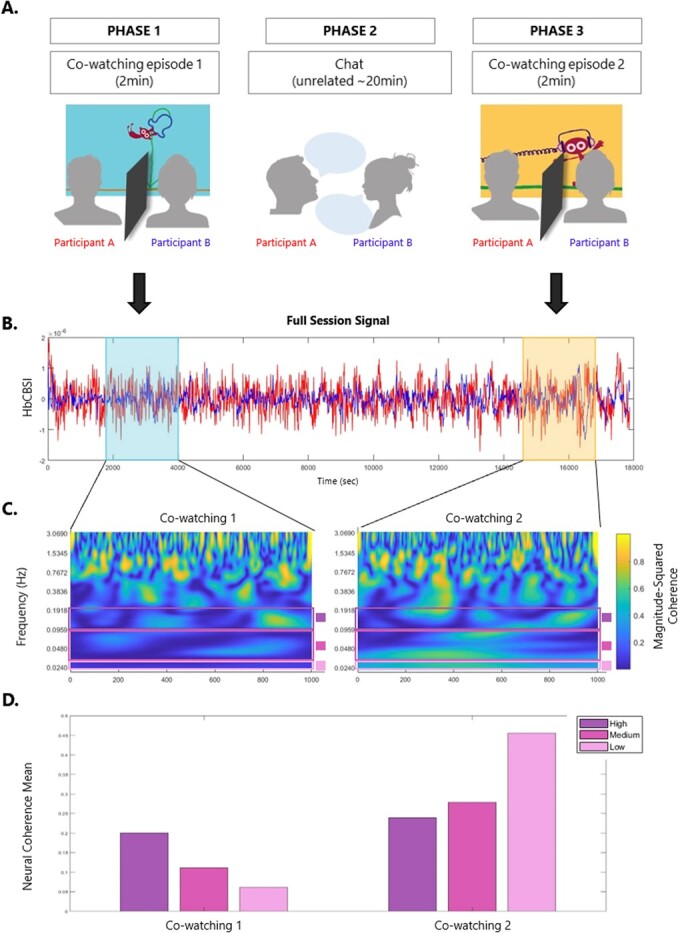
**Example of data processing streamline for one dyad (and one channel/ROI)** – A. participants seat next to each other and watch an episode of the BBC series ‘Dipdap’ (Phase 1). After watching one episode, the two participants engage in a social interaction task, when they chat about unrelated topics (Phase 2). They than watch another – novel - episode of Dipdap (Phase 3). During co-watching (phase 1 and 3), a separator ensures that participants do not engage in any form of communication. The two Dipdap episodes were randomly allocated to phase 1 or 3 (counterbalanced across dyads). They are all non-verbal, self-contained, identical for duration and comparable in terms of audio/visual features. B. Full session Nirs Signal (HbCBSI) plotted for participant A (red) and participant B (blue). Nirs signal during each video cowatching is highlighted. C. Wavelet coherence spectrogram for video 1 and video 2. Bars show the frequency of interest used in analysis. D. Bars plot of the mean for the three frequencies of interest (High: 0.1-0.2 Hz, Medium: 0.03-0.1 Hz, Low: 0.02-0.03 Hz) for video 1 and video 2. Data plotted in B., C. and D. belongs to the same dyad.

Participants sat next to each other facing a screen (Fig. 2*A*). Once the NIRS cap was placed and recording locations localised, the experiment started. Participants first watched one episode of the BBC Dipdap animated series (Phase 1), then chatted about unrelated topics for about 20 minutes (Phase 2), before watching another (new) Dipdap episode (Phase 3). Each episode lasts 2 minutes and shows the adventures of Dipdap, an animated puppet who has to face a series of challenges drawn on the screen by a line. The episodes are all non-verbal and can be watched in any order as they are all self-contained. They are particularly useful to engage the watcher’s imagination, as one follows the drawing line creating new and surprising scenarios for the puppet. The two episodes, ‘*Balloon*’ (no.7) and ‘*Headphones*’ (no.38), were selected randomly from the full list of episodes (available at https://www.bbc.co.uk/iplayer/episodes/b00xgpj9/dipdap?page=1), and were presented in a counter-balanced order over phase 1 (pre-conversation) and phase 3 (post-conversation). During the co-watching parts (phase 1 and 3), a separator was placed in between the two participants, ensuring they did not engage in any social communication *during* the presentation of the episode. In fact, it has been shown that even eye-contact without verbal exchange modulates brain synchrony in dyads [17]. From an ecological point of view, this would resemble being at the cinema, where the dark environment prevents one from seeing their friends, although they may be aware of their presence next to them. Similarly, in this experiment participants could not see their partner’s face but could perceive their presence next to them as they were sitting next to each other. During the social interaction part (Phase 2, conversation phase), participants undertook another experiment where they engaged in a face-to-face semi-structured conversation for about 20 minutes, during which they shared facts about novel items (e.g. exotic animals, musical instruments etc) to each other. Specifically, each participant alternatively played the role of the teacher (sharing facts previously learned) and the role of the learner (listening to the teacher’s description and memorising the facts). During the conversation phase, participants were instructed to memorise facts from each other (as part of another experiment). None of the participants ever mentioned the cartoon episode during the conversation phase.

**Figure 3:**
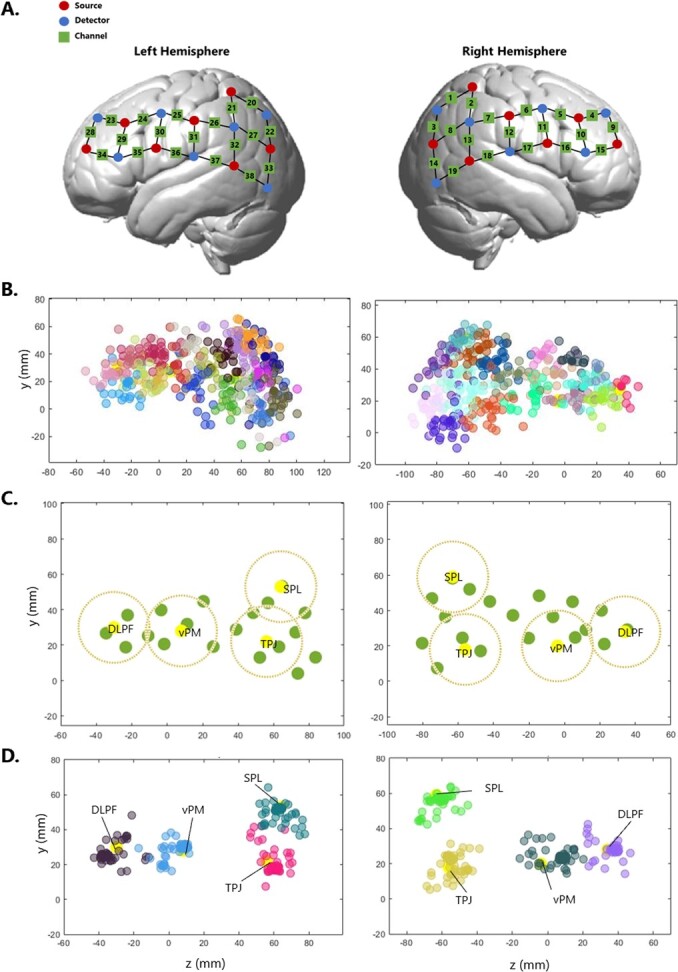
**From headset probe locations to Region of Interest** – A. NIRS headset configuration. Optodes are divided by 7 sources and 7 detectors per hemisphere, spreading from parietal to frontal regions. This configuration forms 19 channels per hemisphere, for a total of 38 channels per participant. B. Channel localization in standard space. Channels (1-38) are plotted, each assigned to one colour, over the whole sample. C. 8 functional ROIs are plotted in yellow, 4 in each hemisphere. Green dots are channels for one participant. For each ROI, the closest channel to the centre would be assigned and contribute with data. No more than one channel would contribute to each ROI per participant. To be assigned to an ROI, channels must be located within the area marked by the dark yellow dotted line around that ROI centre (radius 2cm). D. Channels plotted after being assigned to one of the 8 ROIs. Each colour represent one ROI. DLPF = Dorso-Lateral Pre-Frontal cortex; VPMC = Ventral Pre-Motor cortex; TPJ = Temporo-Parietal Junction; SPL = Superior Parietal Lobe.

### fNIRS signal acquisition

Hemodynamic signals were acquired using a 56 optode (28 sources and 28 detectors, split between two heads) continuous wave NIRS system (Shimadzu LABNIRS, Kyoto, Japan) with three wavelengths of light (780, 805 and 830 nm). Each participant in a dyad had the same distribution of 38 channels over both hemispheres (7 source and 7 detectors per hemisphere, Fig. 3*A*), with a source-detector distance of 3 cm. Before starting the recording, data quality was optimized by adjusting the detector’s gain to maximize signal intensities and improving the optical coupling between the optodes and the scalp (e.g. by moving the hair away from underneath the optodes). Data was collected at a sampling frequency of 8.33 Hz.

### fNIRS data pre-processing

**Figure 4:**
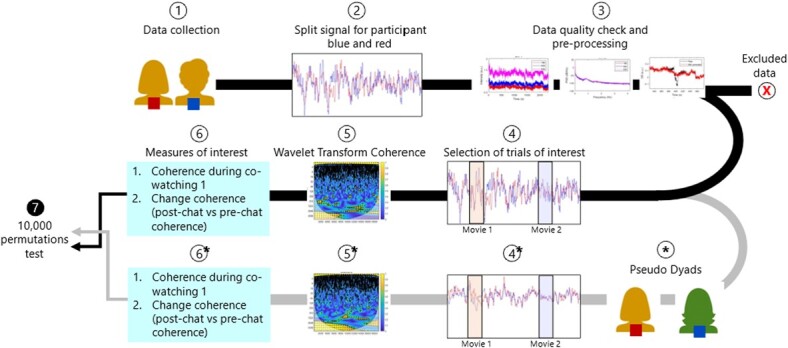
**Data analysis pipeline.** Schematic of the data analysis pipeline for one dyad. Step 1: data is collected from a real dyad (two people visiting the lab together); Step 2: nirs signal from one session is split between the participant-red‘s signal and the participant-blue‘s signal; Step 3: nirs signal for each channel goes through pre-processing and visual quality checks (see methods), after which it is either included or excluded; Step 5: trials of interest (e.g. ‘Movie 1’, see Methods) are extracted from the full session timeseries; Step 5: wavelet coherence analysis is run between participants separately for each trial to obtain a measure of brain synchrony during that trial. Step 6: measures of interest are computed, namely i) coherence during co-watching movie 1 and ii) coherence change after the conversation phase (coherence co-watching movie 2 minus coherence co-watching movie 1). Step 4, 5 and 6 are also identically executed for pseudo dyads (*). Pseudo dyads are computed on the basis of some pre-assigned characteristics to match real dyads on all experimental factors (e.g. trial order, participant colour allocation, see Methods). Step 6: 10,000 permutations were run for each measure of interest separately, between values obtained from the real dyads and values obtained from the pseudo dyads.

The full data processing pipeline is illustrated in Fig. 4. Raw intensity data from all optodes of both participants were first visually inspected to identify noisy channels. Specifically, channels were excluded if no heart beat oscillation was visible in the frequency spectrogram, or if light saturation artefacts and/or large motion errors were present (Fig. 4, step 3). After the data quality check and exclusion, on average each channel had 44 data points (out of 54 participants, min =31; max = 53). For the included channels, raw intensity signals at the 3 wavelengths were pre-processed using the Homer2 toolbox. In particular, intensity data were converted into changes in optical density (function: hmrIntensity2OD). Optical densities were then corrected for motion artifacts using the wavelet-based method (function: hmrMotionCorrectWavelet, iqr = 1.5) and band-pass filtered in the range [0.01 0.4] Hz (5^th^ order Butterwort filter, function: hmrBandPassFilt). Changes in HbO2 and HbR were calculated using the modified Beer–Lambert law assuming a fixed DPF of [6 6 6] (function: hmrOD2Conc). HbO2 and HbR were then combined into the ‘activation signal’ through the CBSI approach (Cui et al. 2012, Burgess et al. 2022).

### Channel to regions of interest (ROIs) allocation

We used a Polhemus Electromagnetic Tracking system (Liberty, Polhemus) to localise each channel in real space. Fig. 3*B* shows the location of each channel across the whole sample (one colour per channel). The variability between participants is visibly large. Such location variability across participant is not unique to our study, but rather a very common issue in studies using fNIRS (Zimeo Morais et al., 2018). This is usually not addressed, and it is commonly assumed that each probe falls in the same location across participants. However, Fig. 3*B* shows how this is not a safe assumption to make. In the section below, we outline some steps we took before data analysis to minimize the negative effects of the variability in probe locations in our data.

#### From real to standard space

Before starting fNIRS data acquisition, we collected 3D space coordinates of the location of each optode from all participants, using a Polhemus Electromagnetic Tracking system (Liberty, Polhemus). We then converted these coordinates from real space (specific to the individual) to Montreal Neurological Institute (MNI) space (where individual locations can be compared), using the NIRS SPM-12 toolbox (Tak et al. 2016). To make sure that the location of each optode was registered correctly, we performed a check on each one of them within each participant: to be classified as correctly registered, we tested the assumption that each optode should be located between 2.5 and 3.5 cm from any neighbour optode (given the 3 cm distance on the cap configuration and taking into account errors in the measurements obtained through the 3D digitizer due to environmental electromagnetic interferences). When this assumption was met, the channel location was computed as the MNI coordinates of the middle point of two adjacent optodes (reflecting the original head configuration, Fig. 3*A*) for each participant. When an optode location was clearly off the standard grid (distance from neighbour optodes was either <2.5 cm or > 3.5 cm), the MNI coordinates for that optode were discarded and the location of any channel forming from the mis-located optode(s) was computed based on the mean MNI coordinate of well-located optodes. For participants where either more than 50% of optodes were mis-located (N = 7) or Polhemus registration failed all together (N = 2), the mean MNI coordinates were used to compute all channel locations.

At the end of this process, every channel in every participant has an MNI coordinate as shown in Fig. 3*B*. This allowed us to compare channel locations across our sample. Noticeably, there is still substantial individual variability in the locations of each channel.

#### From channel to regions of interest

Using the database neurosynth (https://neurosynth.org/), we identified functional ROIs that would be potentially engaged during this study and that were of interest for our hypotheses. Specifically, we identified *x, y,* and *z* coordinates for left and right hemisphere for the following terms: working memory - planning - DLPFC [1091 studies]; speech production [107 studies]; speech comprehension [424 studies]; TPJ/theory of mind [181 studies]; visual cortex [488 studies]; parietal - memory retrieval/episodic memory/joint attention [324 studies]. For each term, the area with the highest activation was identified as the ‘centre’ of the ROI. When terms produced extensive cortical activation (e.g. language terms over left hemisphere), an extra ‘centre’ was selected. A total of 18 centres of interest where identified across the two hemispheres.

To check that these ROIs were in line with the headset configuration, and that we had enough data points for each ROI, we plotted the *x, y,* and *z* coordinates for each centre of interest along with the mean MNI for all channels. We then generated a spheres for each ROI, having as centre the ROIs centre, and a radius of 2 cm. From here, all ROIs that have less than 44 data points falling within the 2 cm radius sphere were excluded. This threshold has been chosen so that our ROIs reflected the distribution of our dataset (44 was the average number of data points available for each channel after the quality check, see ‘fNIRS data pre-processing’, section 2.4).

After this process, 8 ROIs were considered for the final analysis, 4 for each hemisphere: Dorso-Lateral Pre-Frontal cortex (DLPF), ventral Pre-Motor cortex (vPM), Temporo-Parietal Junction (TPJ), and Superior-Parietal Lobe (SPL; Fig. 3*C*). The MNI coordinates for all ROIs are reported in Table 1. For each participant, each channel was assigned to the closest ROI, and in turn it was checked that each ROI was receiving signal from the closest channel (e.g. if channel 27 was *the* clos*est* channel to SPL, but in turn it was clos*er* to TPJ, it was assigned to TPJ, and the next closest channel to SPL was instead assigned to SPL). All channels contributing to any ROI met the assumption that a) were not more than 2 cm from the ROI centre and b) they each contributed to just one ROI. At the end of this process, each ROI had 54 allocated channels from 54 participants, apart from the left SPL which had 41 channels (Fig. 3*D*).

### Pseudo-dyads

The aim of this study was to investigate neural synchrony as a potential marker for social cognition. In order to distinguish the neural coupling arising from simply being exposed to the same sensory experiences (e.g. watching a video), from neural synchrony arising from social cognitive processes, pseudo-dyads were created (Fig. 4). Pseudo-dyads were computed respecting the same experimental characteristics of real dyads, including video presentations and condition order. For example, if real dyad 2 (formed by participant blue 2 and red 2) and real dyad 25 (participant blue 25 and red 25) had both watched the *Balloon* Dipdap episode first, and the *Headphones* Dipdap episode last, they will form one real-dyad subgroup. Then, all possible combination of pseudo-dyads were computed within each real-dyads sub-group (e.g. pseudo-dyads 1: blue 2 and red 25, pseudo-dyad 2: blue 25 and red 2). These ensured that pseudo-dyads would be exactly the same as real-dyads in all aspects of the experimental procedure, apart from the main factor of interest, i.e. having participated in the experiment *together.* A total of 198 pseudo-dyads were created.

### Data analysis

#### Wavelet coherence analysis

Our brain synchrony measure was obtained by running a wavelet coherence analysis on the correlation-based signal improvement (CBSI) using the MATLAB R2020b function *wcoherence* (Fig. 2*C*). Focusing on the CBSI signal has been proven to be a robust way to include information from both oxygenated (HbO) and deoxygenated (HHb) haemoglobin signal from fNIRS (Hakim et al., 2022). The main strength of wavelet coherence analysis over more simple correlation analyses is that it takes into account both the temporal and frequency characteristics of the two signals (Grinsted et al., 2004; Müller et al., 2004). Wavelet coherence was calculated for each ROI within both real and pseudo dyads, for each trial separately (video 1 and video 2). This gave the spectrogram for each dyad in time-frequency space.

Based on a systematic investigation of wavelet coherence transform for fNIRS data, we have focused on frequency ranges that reflect fluctuations of hemodynamic activity [29]. Frequencies below 0.01 Hz are generally linked to noise, such as instrumental noise or vascular endothelial regulations [21, 27] and therefore were not included in the analysis. Similarly, frequencies above 0.2 Hz are too fast for hemodynamic-related activity [21]. In fact, the hemodynamic response is ‘slow’, peaking roughly 5 seconds after neural activity.

We have examined inter-brain coherence at different time scales and in doing so we have considered ranges rather than single frequencies to account for between-subjects frequency variations [30]. Previous works have shown that interpersonal synchrony is spread across multiple frequencies [18] and that frequency components in the range 0.01-0.1 Hz reliably reflect components of neuronal origin [31]. Specifically, we selected three frequency bands of interest, namely high (0.1-0.2 Hz, i.e. 5-10 sec period), medium (0.03-0.1 Hz, i.e. 10-30 sec period), and low (0.02-0.03 Hz, i.e. 30-60 sec period; Fig. 2*C* and 2*D*). This decision was informed by both a general agreement in the literature that different frequencies in hemodynamic rhythms are reflecting different cognitive processes (Cannon et al., 2014; Ward, 2003), and more specifically previous studies looking at brain coherence in social interaction contexts (Cui et al., 2012).

The high frequency range above 0.1 Hz was chosen to investigate if any synchronization occurred at higher frequencies. We believe that this would be plausible as previous fMRI studies suggested that there can still be neuronal contributions to the hemodynamic/BOLD signal above 0.1 Hz [7] and that these can reveal new properties of the brain organization in response to external inputs [6]. Interpersonal synchronization above 0.1 Hz has also been reported in other hyperscanning studies [18].

The medium frequency band was chosen to include the time scale of typical hemodynamic responses to a single event, therefore highlighting the synchronization of transient neural activation across the two brains [23].

The low frequency band was chosen to investigate if any synchronization between spontaneous neuronal activities is observed between participants and also between more sustained hemodynamic activity within our long task cycle (2 minutes).

Once all real and pseudo-dyads had a coherence index for each ROI separately for the video 1 and video 2 trial, the mean in brain coherence across videos was also computed for each ROI. This gave us a general measure of brain coherence during video co-watching. In addition, in order to investigate whether social interaction was responsible for any *change* in brain coherence between participants, the brain coherence difference between video 2 and video 1 was also computed. Therefore, our final matrix had 27 dyads (real, or 198 pseudo) x 2 brain coherence measures (mean and change) x 3 frequency bands (high, medium and low) x 8 ROIs (DLPF left and right, vPM left and right, TPJ left and right, and SPL left and right).

#### Permutation testing

In order to answer the question of whether i) being physically in the same room and ii) having a conversation would drive brain coherence, above and beyond what would be explained by simply processing the same stimulus (e.g. watching the same video), 10.000 permutations were computed between real and pseudo dyads (Fig. 4, step 7). Permutation test has been proved to be a robust analysis tool to control for risk of type-1 error in multiple comparisons (Lage-castellanos et al., 2010; Pesarin, 2001).

The logic here is that real and pseudo dyads share the same features (they all watched the same videos, in the same order, and participated in the same experiments, in the same room), a part from the one factor of interest: pseudo dyads, in contrast to real dyads, did not experience those things *together,* and did not have a conversation *between each other*.

We run two permutation analyses (these refers to the two measures of interest in Fig. 4, step 6). For the first one, we used the input statistic of brain coherence during co-watching phase 1 (pre-conversation), computed for each real dyad and each pseudo dyad. We then calculate a t-statistic for the difference between real and pseudo dyads. We permuted the labels on the data (real or pseudo) 10 000 times and calculated a distribution of t-statistics. We then tested if the true t-stat was different to the permuted t-stat (Fig. 4, step 7). This would help us answer the questions of whether real dyads would be associated with more brain coherence beyond what would be expected by simply being exposed to the same stimuli (pseudo dyads).

For the second permutation analysis, we calculated the input statistic as the brain coherence *change* as a function of conversation (i.e. brain coherence co-watching 2 [post-conversation] *minus* brain coherence co-watching 1 [pre-conversation], computed for real dyads and pseudo dyads. We then followed the same procedure as above: t-statistic was calculated for the difference between real and pseudo dyads. The true t-statistic was then compared to t-statistic from 10 000 samples of permuted labels. This would help us answer the questions of whether social interaction would lead to change in brain coherence beyond what would be expected by merely engaging in a talking task (i.e. detached from your interlocutor, pseudo-dyads).

For each of the two measures of interest (co-watching 1 and pre−/post-conversation change), 10 000 permutations were repeated separately for the 8 ROIs and for the three frequency bands, for a total of 240 000 permutations per measure (Lage-castellanos et al., 2010; Pesarin, 2001).

## RESULTS

**Figure 5:**
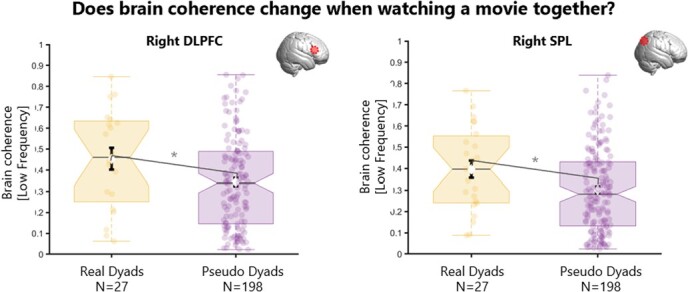
**Results for brain coherence during movie co-watching (phase 1) for real and pseudo dyads.** Boxplots showing the distribution for real dyads (yellow) and for pseudo dyads (purple) of the brain coherence during co-watching phase 1 (before conversation). There was significantly more coherence (low frequency 0.02-0.03 Hz) in real vs pseudo dyads over right DLPFC (right panel) and right SPL (left panel). DLPFC = dorso-lateral pre-frontal cortex; SPL = superior parietal lobe.

**Figure 6:**
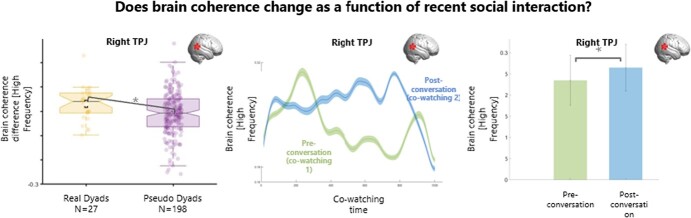
**Results for brain coherence change after engaging in conversation.** Plots of brain coherence difference between co-watching 2 (post-conversation) and co-watching 1 (pre-conversation). Left panel: boxplots of the distribution of brain coherence difference across co-watching phases for real (yellow) and pseudo (purple) dyads. After a conversation, there was significantly more coherence in real vs pseudo dyads over right TPJ. Right panel: brain coherence for real dyads (sample mean) during co-watching preconversation (phase 1) and co-watching post-conversation (phase 2) over session duration. ^*^p<.05. SPL = Superior Parietal Lobe, TPJ = Temporo-Parietal Junction.

Full results are reported in Table 2 and 3 for all ROI and all frequency bands. Main findings are presented in Fig. 5 and Fig. 6. None of these results survived correction for multiple comparisons.

**Table 2:** Results for brain coherence during co-watching in phase 1

**Frequency band**	**Region**	**Real dyads mean (sd)**	**Pseudo dyads mean (sd)**	**Observed diff.**	***p-value***	**Effect size**	**Confidence Interval**	
High (.1 - 0.2 Hz)								
	DLPF right	.26 (.06)	.25 (.07)	.01	.51	.07	−.33	.47
	DLPF left	.26 (.06)	.26 (.06)	.00	.98	.06	−.34	.46
	vPM right	.23 (.05)	.24 (.06)	−.016	.19	−.32	−.72	.08
	vPM left	.27 (.06)	.25 (.07)	.02	.12	.24	−.16	.64
	TPJ right	.23 (.06)	.25 (06)	−.02	.13	−.31	−.71	.09
	TPJ left	.24 (.06)	.26 (.07)	−.01	.37	−.27	−.68	.13
	SPL right	.24 (.08)	.25 (.07)	−.01	.52	−.12	−.52	.28
	SPL left	.22 (.06)	.24 (.08)	−.02	.09	−.30	−.70	.11
Medium (.03 - 0.1 Hz)								
	DLPF right	.24 (.09)	.23 (.09)	−.00	.95	−.22	−.63	.18
	DLPF left	.22 (.09)	.24 (.08)	−.01	.57	−.52	−.93	−.12
	vPM right	.25 (.09)	.24 (.08)	.00	.81	.03	−.38	.43
	vPM left	.24 (.09)	.24 (.08)	.01	.68	−.19	−.59	.21
	TPJ right	.22 (.08)	.24 (.08)	−.02	.14	−.57	−.98	−.17
	TPJ left	.24 (.07)	.24 (.07)	.00	.87	−.22	−.62	.18
	SPL right	.24 (.07)	.25 (.10)	.00	.92	−.23	−.63	.17
	SPL left	.20 (.07)	.21 (.08)	−.01	.54	−.49	−.89	−.08
Low (.02 - 0.03 Hz)								
	**DLPF right**	**.41 (.22)**	**.37 (.24)**	**.11**	**.04**	**.19**	**−.21**	**.59**
	DLPF left	.30 (.21)	.31 (.22)	−.00	.95	−.04	−.44	.36
	vPM right	.33 (.21)	.33 (.22)	−.01	.88	−.01	−.41	.40
	vPM left	.33 (.17)	.33 (.20)	.02	.72	−.01	−.41	.39
	TPJ right	.30 (.21)	.28 (.20)	.03	.54	.12	−.28	.53
	TPJ left	.30 (.19)	.31 (.20)	.00	.94	−.04	−.44	.36
	**SPL right**	**.38 (.21)**	**.31 (.20)**	**.10**	**.03**	**.34**	**−.06**	**.74**
	SPL left	.29 (.19)	.28 (.19)	.02	.71	.05	−.35	.45

Results from 10 000 permutations test for brain coherence between real and pseudo dyads. Permutation statistic used was the brain coherence during co-watching in phase 1 (before conversation).

**Table 3:** Results for brain coherence difference after conversation

**Frequency band**	**Region**	**Real dyads mean (sd)**	**Pseudo dyads mean (sd)**	**Observed diff.**	***p-value***	**Effect size**	**Confidence Interval**	
High (0.1-0.2 Hz)								
	DLPF right	−.017 (.08)	−.005 (.07)	−.02	*.44*	−.17	−.57	.23
	DLPF left	−.004 (.08)	−.003 (.08)	.00	*.96*	−.01	−.41	.39
	vPM right	.016 (.06)	.001 (.09)	.02	*.37*	.18	−.22	.59
	vPM left	−.018 (.07)	0 (.08)	−.02	*.30*	−.21	−.62	.19
	**TPJ right**	**.030 (.07)**	**−.008 (.09)**	**.04**	***.03***	**.44**	**.03**	**.84**
	TPJ left	.006 (.09)	−.002 (.08)	.01	*.66*	.09	−.31	.50
	SPL right	.007 (.08)	−.004 (.07)	.01	*.50*	.14	−.26	.54
	SPL left	.019 (.07)	.01 (.08)	.01	*.68*	.11	−.29	.52
Medium (0.03-0.1 Hz)								
	DLPF right	−.003 (.09)	−.007 (.10)	.00	*.86*	.03	−.37	.43
	DLPF left	−.016 (.11)	.005 (.10)	−.02	*.31*	−.20	−.60	.20
	vPM right	−.004 (.13)	.001 (.11)	−.01	*.82*	−.05	−.45	.35
	vPM left	−.004 (.12)	−.007 (.11)	.00	*.91*	.02	−.38	.42
	TPJ right	.031 (.13)	−.01 (.11)	.04	*.08*	.37	−.03	.77
	TPJ left	.023 (.10)	−.003 (.10)	.03	*.24*	.25	−.15	.65
	SPL right	−.005 (.10)	−.006 (.10)	.00	*.97*	.01	−.39	.41
	SPL left	.034 (.09)	.009 (.09)	.03	*.26*	.27	−.13	.67
Low (0.02-0.03 Hz)								
	DLPF right	−.056 (.25)	.002 (.24)	−.08	*.26*	−.24	−.64	.17
	DLPF left	−.028 (.29)	−.003 (.31)	−.03	*.69*	−.08	−.48	.32
	vPM right	.015 (.32)	.009 (.30)	.01	*.93*	.02	−.38	.42
	vPM left	.048 (.26)	.002 (.28)	.05	*.42*	.17	−.23	.57
	TPJ right	.054 (.30)	.043 (.27)	.01	*.85*	.04	−.36	.44
	TPJ left	.112 (.29)	.006 (.26)	.11	*.07*	.40	0	.80
	SPL right	−.052 (.27)	.034 (.26)	−.10	*.11*	−.33	−.73	.07
	SPL left	.088 (.26)	.041 (.23)	.05	*.44*	.20	−.20	.60

Results from 10 000 permutations test for brain coherence difference across co-watching phases between real and pseudo dyads. Permutation statistic used was the difference brain coherence across videos (co-watching phase 2 - co-watching phase 1).

### Does brain coherence change when watching a movie together?

Brain coherence during co-watching of video 1 (phase 1) was significantly higher in real dyads compared to pseudo dyads over right DLPFC (t = 0.11; *p = 0.04*) and right SPL for the low frequency band (t = 0.10; *p = 0.03*). In other words, being physically in the same room during co-watching (real dyads) was associated with *more* brain coherence over the DLPFC and SPL in the right hemisphere, compared to what would be expected on average for processing the movie (pseudo dyads). No difference was found in brain coherence (in any frequency bands) for all other ROIs between real and pseudo dyads.

A preliminary analysis used the mean of brain coherence across co-watching phases (i.e. pre- and post-conversation). This however may have been confounded by conversation-related effect. For transparency, results from this previous analysis are reported in supplementary materials.

### Does brain coherence change as a function of recent social interaction?

The difference in brain coherence between co-watching phases (coherence video 2 – coherence video 1) was significantly higher in real dyads compared to pseudo dyads over right TPJ for the high frequency band (t = 0.04, *p = 0.03;*Fig. 2). In other words, brain coherence for the high frequency band over the right TPJ during subsequent co-watching of a novel video was higher between two interlocutors (real dyads), than between two people who did *not* have a chat *with each other* (pseudo-dyads).

Noticeably, as we discussed earlier, real and pseudo dyads differ also on the basis of familiarity: that is, real dyads had a conversation with each other *and* knew each other, while pseudo dyads did not. Therefore, these results may reflect having a conversation, being familiar with someone, or an interaction between these two factors. In order to confirm that the effect we observed is due to our experimental manipulation (having a conversation), we conducted a simple t-test of the brain coherence of real dyads only before and after they had a conversation (i.e. during co-watching in phase 1 and co-watching in phase 2, Fig. 6). Results confirm that having a conversation increased brain coherence during later co-watching over the right TPJ for the high frequency band (t(26) = 2.25, p = 0.03, CI [.06 - 0.003]).

## DISCUSSION

In this study, we asked whether social factors (e.g. co-presence and face-to-face interaction) could specifically contribute to brain synchrony above what would be expected by simply watching the same video. We used hyperscanning to measure neural responses during movie co-watching in familiar pairs (dyads) before and after they engaged in a conversation. We report two main findings: first, over the right hemisphere, real dyads showed *increased* brain synchrony over Dorso-Lateral Pre-Frontal cortex (DLPFC) and Superior Parietal Lobe (SPL) during co-watching, compared to pseudo dyads (who had never seen each other and watched the same movie at different times). Second, real dyads who engaged in conversation showed *increased* synchrony over right TPJ during subsequent novel movie co-watching, and significantly more than what was observed in pseudo dyads. We discuss each of these findings in turn.

First, when comparing brain-to-brain synchrony between real dyads and pseudo dyads during the first co-watching phase, we found that real dyads showed greater synchrony over DLPFC and SPL in the right hemisphere. Increased neural synchrony over DLPFC during co-watching is consistent with previous studies showing that familiar pairs of people attending to stimuli together showed increased synchrony compared to both unfamiliar (shuffled) pairs and solo-experience [1–3, 10]. DLPFC has been found to play a crucial role in social bonding [4], emotional resonance between people sharing painful experiences [20] and in regulating in-group dynamics [26]. It is possible that our pairs of participants, being both familiar to each other and sharing the same experience of watching a movie together, engaged in some co-regulation which resulted in increased synchrony over this region. Similarly, sharing the same physical environment may have increased synchrony of their proprioceptive systems, resulting in greater synchrony over the right SPL [11, 22], compared to pseudo dyads who did not share the same spatio-temporal environment. Frontal–parietal circuit has also been found to play a key role in constructing mental models of self- and other-representations [5, 9, 14], which may explain why these regions showed greater synchrony between people who were physically sharing an experience together.

Importantly, real dyads differ from pseudo dyads for two main features: they are familiar with their partner *and* they *co-*experienced the movie watching in time and space. Therefore, contrasting our real pairs with fake pairs *de facto* resulted in contrasting familiar versus unfamiliar pairs. The fact that participants knew each other was dictated by the fact that data was collected during the covid-19 global pandemic, where UK government only allowed people within the same household to meet and interact face-to-face. We acknowledge this is a major limitations of this study and future work should use similar paradigm to compare familiar versus stranger dyads. While the contribution from these two factors (familiarity and co-presence) is difficult to disentangle here, we can make some speculations on how these may have modulated synchrony in this study.

Greater brain coherence between familiar pairs (real dyads) may reflect emotional attunement (Nummenmaa et al., 2012), shared psychological perspectives (Lahnakoski et al., 2014) and social closeness (Wolf & Tomasello, 2020) typical of intimate relationships. However, in contrast to previous studies on neural alignment, this study measured brain activity from each pair *simultaneously*. It may be that co-experiencing movie-watching would additionally modulate brain synchrony in real pairs (beyond familiarity), in ways that are not possible when watching the same movie alone. Here, we therefore refer to *stimulus-driven brain coherence* to distinguish cognitive processing during co-experiences (like in this study), from neural alignment of cognitive processing happening solo (like in Parkinson et al., 2008). Sharing a physical environment activates processes of self-location and vestibular regulation with reference to the external world (Ionta et al., 2011): physical proximity may therefore engage a series of computations that may align the brains of people immersed in the same spatial–temporal context (Hamilton, 2020), in ways that do not occur in alone experiences. Mechanisms of familiarity and co-presence are unlikely to be mutually exclusive and possibly modulate brain-to-brain dynamics in tandem. Future studies should disentangle the effect of physical proximity from familiarity in aligning brain activities during sensory processing, by contrasting familiar and unfamiliar pairs (or groups) and directly comparing solo experiences with shared experiences.

Our second main finding was an increased level of brain coherence over right TPJ after face-to-face social interaction (difference in brain coherence between co-watching post-conversation and co-watching pre-conversation). In other words, *after* people engaged in conversation, their brain response to a novel movie watching was *more similar* compared to what was observed before the conversation, and increased to a significantly greater degree in real pairs compared to pseudo pairs. We refer to this effect as *socially-driven brain coherence*. There are two important points to highlight here: first, both real dyads and pseudo dyads engaged in conversation, but crucially only real dyads conversed with one another, while pseudo dyads conversed with someone else (i.e. their real partner rather than the one forming the pseudo pair). Therefore, the observed effect cannot be explained by simply engaging in any social exchange, but specifically arises from *interaction with one another*. The second important element to consider is that conversation did not touch upon the content of the movie at any point, and the movie presented after the conversation was a novel one. This means that the observed increase in neural coherence cannot be interpreted as reflecting explicit consensus over a specific instance (e.g. one particular movie), but rather suggests that social interaction may support the development of general common ground and shared-understanding for later events, in ways that are not attached to a specific paradigm or context.

These results are consistent with previous studies showing conversation-related neural similarity between people (Sievers et al., 2020). Importantly, they go beyond existing literature by demonstrating that social interaction distinctly contributes to increase synchrony over right TPJ between people co-experiencing later events. The right TPJ has been found to be associated with shared-understanding of external reality (Nguyen et al., 2019; Salazar et al., 2021; Yeshurun et al., 2017), as well as being heavily involved in social processes including mentalising (Molenberghs et al., 2016) and face-to-face conversation (Cañigueral et al., 2021). The non-verbal Dipdap episodes used in this study are likely to elicit internal narratives of what is about to happen next, as a puppet is challenged by an imaginary line creating new unpredictable adventures. Here we show that neural response in the right TPJ becomes more similar between people who just engaged in social interaction, even to later unpredictable events. Future studies should further test this hypothesis by comparing brain coherence with explicit individual reports of their interpretation of the new event.

While we cannot be sure about the exact mechanisms behind the observed increase in brain coherence after conversation, one can suggest some speculations. It may be that the common ground and shared-understanding built over the conversation extended beyond it to immediately later events. Studies on mimicry suggests that during conversation people tend to naturally mirror their interlocutors body posture, speech rate, and even word-choice, with the ultimate goal of alignment of high-level mental representation (Garrod & Pickering, 2004, 2009). This effect has been shown to last also after the conversation event (Richardson et al., 2007). In the present study, during the conversation phase, participants engaged in a ‘teacher-learner’ interaction (alternating roles), when they were sharing information to each other about novel items (e.g. an exotic animal). In teacher-learner interactions, the primary objective is the efficient transfer and reception of information, fostering the alignment of high-level mental representations. If this interpretation is correct, synchrony would be observed not only at the neural level but also for other physiological signals (e.g. breathing, (Konvalinka et al., 2023; McFarland, 2001), and eye-movements (Richardson et al., 2007; Richardson & Dale, 2005), and would also be stronger in dyads where such signals coupled more *during* the conversation. Future studies should test this hypothesis.

Our results also suggest that brain coherence is affected by social processes differently across different frequency bands. We hesitate to make strong conclusions about the specific underlying cognitive mechanisms subserving these different frequencies, as these are difficult to interpret. Previous fNIRS studies have mainly looked at one frequency band only (Cui et al., 2012; Lu & Hao, 2019), and more direct investigations are needed to test different frequency components in relation to specific cognitive processes. Also, fNIRS has a relatively slow temporal resolution and cannot measure changes happening faster than the hemodynamic response (~5 seconds). Future work should combine different neuroimaging modalities to investigate a broader range of frequency components. However, the present findings suggest that different frequency bands may reflect difference in neuronal rhythms, possibly mirroring the complexity of spatio-temporal dynamics in social interaction, in line with previous work on both social and non-social processing (Cannon et al., 2014; Ward, 2003).

Using fNIRS in this study has allowed us to investigate real-world interaction in ways that other neuroimaging modalities would have not made possible (e.g. fMRI). Specifically, it provided a way to study *real-time brain synchrony* to investigate questions which have only been considered in terms of inter-subject correlation so far. However, the downside of this includes relatively poor spatial resolution. By re-allocating channels to specific ROIs based on their MNI coordinates (see methods), we have tried to minimize this limitation. Exact comparisons across studies in terms of neuro-anatomical regions and associated functional processes, especially when data comes from tools with high spatial precision like fMRI, should however still be inferred with caution. Future studies should combine the use of multiple techniques to integrate strengths from different neuroimaging modalities, as well as making use of other technologies to include behavioural and physiological data to disentangle the contribution from different factors in driving brain coherence.

The reported results are novel and hold the potential to stimulate constructive debates on the underlying mechanisms of brain synchrony within various social contexts. We hope that this work will encourage future studies to explore the entire spectrum of social interactivity, ranging from co-presence to face-to-face conversation, and their long-term effects on subsequent interactions. However, the fact that our results would not survive a multiple-comparisons correction, led us exercise caution and refrain from making definitive claims based solely on these present results to explain the phenomenon of brain synchrony during co-watching. By running our analysis with a considerable number of permutations and a large sample of pseudo dyads we have made an attempt to make our results robust, but future studies are needed to confirm our findings.

## CONCLUSIONS

In conclusion, in this study we showed how social interaction can distinctly affect brain response across people in real-time, for later processing of non-social signals (movie watching). We demonstrated how co-experiencing a simple activity like watching a movie can couple brain regions. This possibly reflects mechanisms of internal and external processing, namely how we experience the world within ourselves and with others. Furthermore, we were able to specifically isolate the role of social interaction and show how interacting *with* someone in particular synchronises brain signals for later events. These results have implications for our understanding of social dynamics and how we share experiences and align interpretation with our friends and family in the real world.

## Supplementary Material

Web_Material_kvae006

## Data Availability

Data is openly available (DOI 10.17605/OSF.IO/FYG7R) on OSF repository, accessible at this link https://osf.io/fyg7r/?view_only=2a25b7b8f31c4ba5ace0c137bcd4ef7a.

## FUNDINGS

S.D.F. was supported by Leverhulme award DS-2017-026.
