# Supplementary material for: Having a chat and then watching a movie: how social interaction synchronises our brains during co-watching
Source: Oxf Open Neurosci. 2024 Mar 25;3:kvae006. doi: 10.1093/oons/kvae006 (PMC11069416; doi:10.1093/oons/kvae006)
Supplement: Web_Material_kvae006 [file Cover Letter & reponse to reviewers.pdf › Cover Letter & reponse to reviewers.pdf]

Sara De Felice
Research Associate
Department of Psychology
Downing Place, CB2 3EB
Cambridge, UK

Dear Dr. Izquierdo,

thank you for your response and for considering our manuscript for submission in Oxford Open Neuroscience Journal.

We believe we have addressed all reviewers' concerns. We copy the reviewers' comments below and include a detailed response to each comment. In addition, we would like to bring to your attention that despite none of the reviewers commenting on the title, we have decided to modify it, as we believe the new version better describes our findings and research question. Please do let us know if this is not possible and we will change it to the original version.

Looking forward to hearing from you.

Best wishes,

Sara De Felice (on behalf of all authors)

Reviewer: 1

Comments to the Author

This paper is clearly written and investigates an interesting question: whether mere social interaction (unrelated conversations), is required to increase the brain-to-brain synchrony between pairs of participants when they watch movies before and after the conversations. The fNIRS results showed different levels of neural synchrony between real dyads and pseudo-dyads, as well as increased synchrony between real dyads before and after conversations.

I enjoyed reading this paper overall, but I have a couple major concerns:

- The authors seem to be missing a large body of literature in their review.

+ For example, the authors mentioned that "the *only* attempt to answer the question of whether social interaction modulates ISC is an fMRI study by (Sievers et al., n.d.)". I think this statement is seriously oversimplifying the research in this field, since there are many studies looking at how social interactions affect brain synchrony. A few examples:

Sara De Felice
Research Associate
Department of Psychology
Downing Place, CB2 3EB
Cambridge, UK

- Yun et al. (2012, https://doi.org/10.1038/srep00959): This study demonstrated that pairs of participants showed increased brain synchrony (measured by EEG) after a cooperative task, which involved social interaction without conversations. I believe this study answered the authors' research question in the current paper to an extent.

- Spiegelhalder et al. (2014, https://doi.org/10.1016/j.bbr.2013.10.015) showed increased brain synchrony (fMRI) during verbal interactions.

- Pan et al. (2023, https://doi.org/10.1093/cercor/bhac127) showed triads of participants had increased neural synchronization (fNIRS) after cooperations.

- Kelson et al. (2022, https://doi.org/10.1016/j.neubiorev.2020.09.008) provided a review on EEG/fNIRS hyperscanning studies of verbal communications.

- Tsoi et al. (2022, https://doi.org/10.1111/spc3.12707) also provided a relevant review on some fMRI studies related to this topic.

+ Considering this body of literature, the novel insights provided by the current study seems to be somewhat limited, even though the results of this study are still quite interesting. It might be necessary for the authors to substantially reframe their introduction/literature review part to cover the past research on this topic more comprehensively, and to evaluate their study's contribution accurately.

We thank the reviewer for pointing us towards many interesting studies, which are certainly related to our work. We have now included some of them in our introduction. However, here we are interested specifically in how naturalistic social interaction (through face-to-face unconstrained verbal exchange) would have effect on IBS *afterwards*, during *non-interactive* experiences. When we write "the only attempt to answer the question of whether social interaction modulates ISC is an fMRI study by Sievers et al.", what we mean is precisely that: a study that have looked at IBS *during non-interactive tasks* (e.g. watching a movie), as *a function of *previous* naturalistic social interaction* (e.g. a conversation). As such, we believe the study by Sievers et al. is the closest and the only one – to our knowledge – that attempted to answer the question. The study by Yun et al. (2012) would be relatively close in the question they address and the logic they employ (there is a before and an after phase where synchrony is compared), however there are a number of major things that make it not relevant to answer our question and not very relatable to our results: it is highly artificial in its design (finger tapping), there is no conversation involved in their 'training phase', they do very similar tasks across all phases (finger tapping or just observing each other finger's tip) and finally participants remain in front of each other in all

Sara De Felice
Research Associate
Department of Psychology
Downing Place, CB2 3EB
Cambridge, UK

phases (arguably 'interacting' throughout the different phases in terms of eye-contact and non-verbal communication). We have now rephrased our introduction to include some of the studies suggested by the reviewer, and made the distinction between how social interaction modulates IBS on-line (during interactive tasks) versus later experiences (i.e. post-interaction and during non-social tasks) more explicit.

Text in bold is new:

Several studies have looked at how brain synchrony varies during – and as a function of – social interaction (Czeszumski et al., 2022; Kinreich et al., 2017; Li et al., 2021; Pan et al., 2023; Spiegelhalder et al., 2014; Yun et al., 2012; for some reviews on this see Kelsen et al., 2022; Tsoi et al., 2022). For example, Pan and colleagues found higher inter-brain synchrony across three participants as they analysed an ancient Chinese poem together (cooperative condition) versus solo-sessions (independent condition). In other study, Nguyen et al., (2021) showed that inter-brain synchrony was predicted by turn-taking events during natural conversations between a child and their mother. This literature demonstrates that brain synchrony reflects inter-personal dynamics arising during social interaction. However, less is known about whether and how social interaction modulates brain synchrony during later non-interactive events. In other words, how does having a conversation aligns brain activity of people afterwards, as they engage in later (non-interactive) experiences, e.g. watching a movie?

An *attempt to answer the question of whether* ***having previously engaged in*** *social interaction modulates ISC* ***during later events*** *is an fMRI study by (Sievers et al., 2020).*

- This study compared dyads who were familiar with each other (friends, flatmates or partners) who co-watched movies and had conversations, to pseudo-dyads who were not familiar with each other who did not co-watch or have conversations. It remains quite unclear whether the results arise from pairs being familiar with each other (as found in Parkinson et al., 2018), or them co-watching movies, or them having conversations, or all three. The authors talked about this in their discussions, but much of the introduction and results was still framed such that it seems like the familiarity was not a factor.
+ Ideally, another experiment could be performed with unfamiliar dyads going through the same procedure, so that their results could be compared. I think this would make the paper a lot stronger and much more interesting. Even better, there could be yet another experiment where participants who only co-watched movies while not

Sara De Felice
Research Associate
Department of Psychology
Downing Place, CB2 3EB
Cambridge, UK

engaging in any conversations, so that the three factors could be separated.
+ Alternatively, I think the authors may also try to (substantially) reframe the paper so that the focus is not on comparing between real and pseudo-dyads, but on comparing just the real dyads between their co-watching phase 1 versus phase 2 (i.e., before versus after having a conversation).

We acknowledge the concerns of the reviewer regarding disentangling the source of our effects. In this study we asked two separate questions: first, is co-presence per se affecting IBS during co-watching? And second, does a conversation modulates IBS during later non-interacting co-watching? There are three potential factors modulating IBS in this study: familiarity, co-presence and conversation. With regards to familiarity, the only way to isolate its effect would be indeed to run another experiment, as the reviewer suggested. Unfortunately, as much as we would like to, doing another experiment would not be feasible for us. This study was part of the first author PhD, which is now completed. There are no resources to support a second experiment. Ideally, we would have included only stranger pairs (and that was our original plan), but when covid-19 restrictions were enforced we were obliged to test people from the same household for safety reasons. We now discuss this confound more explicitly in the introduction (new text in bold):

Note that all participants were familiar pairs (e.g. friends, flatmates or partners). This was dictated by the fact that data was collected during the covid-19 global pandemic, where UK government only allowed people within the same household to meet and interact face-to-face. ***We acknowledge this is a major limitations of this study and future work should use similar paradigm to compare familiar versus stranger dyads.***

as well as discussion (new text in bold):

*Importantly, real dyads differ from pseudo dyads for two main features: they are familiar with their partner and they co-experienced the movie watching in time and space. This was dictated by the fact that data was collected during the covid-19 global pandemic, where UK government only allowed people within the same household to meet and interact face-to-face****. We acknowledge this is a major limitations of this study and future work should use similar paradigm to compare familiar versus stranger dyads.*** *While the contribution from these two factors is difficult to disentangle here, we can make some speculations on how these may have modulated synchrony in this study.*

Sara De Felice
Research Associate
Department of Psychology
Downing Place, CB2 3EB
Cambridge, UK

With regards to the effect of co-presence and conversation, we have conducted two further analysis, which attempt to disentangle the effect from these two factors. We discuss these separately.

With regards to how co-presence modulates IBS (question 1), we initially computed the mean of IBS during co-watching phases (co-watching of video 1 and video 2) for both real and pseudo (randomly shuffled) dyads, and conducted permutation testing to see whether there was any difference between real dyads watching a movie together versus pseudo dyads watching a movie with some random person. Our reasoning was that by averaging both videos, our signal would have been more robust to noise (e.g. external environmental factors). However, as reviewers pointed out, the mean across videos would also include some effects from conversation (as video 2 always happened post-conversation). We have therefore decided to replicate our previous analysis comparing real and pseudo dyads only for video 1. This way we got rid of the potential confounding factor of conversation in question 1 (does co-presence alone modulate IBS?). Results from this new analysis reveal significantly more IBS in real dyads over right DLPFC and right SPL. For transparency, we have included results from the previous analysis in Supplementary Materials. We include 'new' results and discussion in the main manuscript (new text in bold). Results section:

Brain coherence during co-watching of video 1 (phase 1) was significantly higher in real dyads compared to pseudo dyads over right DLPFC ($t = .11$; $p = .04$) and right SPL for the low frequency band ($t = .10$; $p = .03$). In other words, being physically in the same room during co-watching (real dyads) was associated with more brain coherence over the DLPFC and SPL in the right hemisphere, compared to what would be expected on average for processing the movie (pseudo dyads). No difference was found in brain coherence (in any frequency bands) for all other ROIs between real and pseudo dyads.

Discussion section:

First, when comparing brain-to-brain synchrony between real dyads and pseudo dyads during the first co-watching phase, we found that real dyads showed greater synchrony over ***DLPFC and SPL in the right hemisphere. Increased neural synchrony over DLPFC during co-watching is consistent with previous studies showing that familiar pairs of people attending to stimuli together showed increased synchrony compared to both unfamiliar (shuffled) pairs and solo-experience (Azhari et al., 2020, 2021, 2022; Durnford et al., 2022). DLPFC has been found to play a crucial role in social bonding (Balconi et al., 2020), emotional resonance***

Sara De Felice
Research Associate
Department of Psychology
Downing Place, CB2 3EB
Cambridge, UK

between people sharing painful experiences (Peng et al., 2021) and in regulating in-group dynamics (Yang et al., 2020). It is possible that our pairs of participants, being both familiar to each other and sharing the same experience of watching a movie together, engaged in some co-regulation which resulted in increased synchrony over this region. Similarly, sharing the same physical environment may have increased synchrony of their proprioceptive systems, resulting in greater synchrony over the right SPL (Ishida & Murata, 2007; Rizzolatti & Sinigaglia, 2010), compared to pseudo dyads who did not share the same spatio-temporal environment. Frontal-parietal circuit has also been found to play a key role in constructing mental models of self- and other-representations (Balconi et al., 2018; Dumas et al., 2010; Konvalinka et al., 2014), which may explain why these regions showed greater synchrony between people who were physically sharing an experience together.

With regards to how conversation modulates IBS (question 2), we initially computed the difference in IBS between co-watching video 1 (pre-conversation) and co-watching video 2 (post-conversation), for both real and pseudo dyads. We then conducted a series of permutations to test whether IBS pre-post conversation was different between real and pseudo dyads. Including pseudo dyads strengthen the results against potential confound related to the environment and/or events contingent to the experimental session (e.g. external sounds, lightening etc). We therefore believe that keeping the pseudo dyads analysis would be important. Equally, we welcome the reviewer suggestion to run another analysis comparing IBS during co-watching of video 1 and video 2, for real dyads only. Results are consistent with our permutation analysis including pseudo-dyads. We report these in our results section (new text in bold) and have also updated our figure 6 to include this analysis:

Noticeably, as we discussed earlier, real and pseudo dyads differ also on the basis of familiarity: that is, real dyads had a conversation with each other and knew each other, while pseudo dyads did not. Therefore, these results may reflect having a conversation, being familiar with someone, or an interaction between these two factors. In order to confirm that the effect we observed is due to our experimental manipulation (having a conversation), we conducted a simple t-test of the brain coherence of real dyads only, comparing IBS before and after they had a conversation (i.e. during co-watching in phase 1 and co-watching in phase 2, Fig. 6). Results confirm that having a conversation increased brain coherence during later co-watching over the right TPJ for the high frequency band ($t(26)=2.25$, $p=.03$, CI [.06 - .003]).

Sara De Felice
Research Associate
Department of Psychology
Downing Place, CB2 3EB
Cambridge, UK

A few other minor points:

- The authors mentioned that a separator was placed in between the two participants while they were co-watching. It seems like this would make the situation somewhat unnatural. How would placing a separator empirically different from placing two participants in separate cubicles? Or neighboring rooms? To what extent would this still count as a "co-watching" experience? I wonder if the authors could justify this in more details (if the only purpose was to prevent social interactions, is it not sufficient to simply tell them to stay still and not talk?).

We wanted to see whether IBS during non-social interaction (co-watching a movie) was affected by social interaction occurring before that event. For this reason, we wanted to minimise any potential confound of the two participants looking at each other, smiling, nodding, giving any sort of feedback to each other during the co-watching event. From an ecological point of view, when we are in cinema watching movies it is all dark around us and we do not really see our friends, but we perceive their presence next to us. Similarly, in this experiment participants could not see their partner's face but could perceive their presence next to them as they were sitting next to each other.

We have now included a more explicit explanation of our choice in the method section (text in bold is new):

During the co-watching parts (phase 1 and 3), a separator was placed in between the two participants, ensuring they did not engage in any social communication during the presentation of the episode. ***In fact, it has been shown that even eye-contact without verbal exchange modulates brain synchrony in dyads (Noah et al., 2020). From an ecological point of view, this would resemble being at the cinema, where the dark environment prevents one seeing their friends, although they are aware of their presence next to them. Similarly, in this experiment participants could not see their partner's face but could perceive their presence next to them as they were sitting next to each other.***

- It would be nice if the authors can elaborate on the participants' conversations and how they made sure it was unrelated to the cartoon. How was it "semi-structured"? Did the experimenter set a topic for the participants?

Thank you for pointing this out. We have now elaborated on this in our introduction (text in bold is new):

Sara De Felice
Research Associate
Department of Psychology
Downing Place, CB2 3EB
Cambridge, UK

After the first co-watching phase, participants engaged in a 20-minute conversation on unrelated topics ***(prompts for the conversation were provided, including sharing facts about exotic animals and musical instruments)****.*

and method section (text in bold is new):

During the social interaction part (Phase 2, conversation phase), participants undertook another experiment where they engaged in a face-to-face semi-structured conversation for about 20 minutes, during which they shared facts about novel items (e.g. exotic animals, musical instruments etc) to each other. ***Specifically, each participant alternatively played the role of the teacher (sharing facts previously learned) and the role of the learner (listening to the teacher's description and memorising the facts). During the conversation phase, participants were instructed to memorise facts from each other (as part of another experiment).***

- The authors may want to check their reference list. For example, Cui et al. seems to be published in 2012, but when the authors cited this paper they mostly wrote 2011 except for one occasion. Also Sievers et al. has been out as a preprint since 2020, and the authors should cite it accordingly (rather than using "n.d.")

We have now implemented these changes.

- There is a typo in the last few lines of page 5 ("than"?).

Thanks, corrected.

Reviewer: 2

Comments to the Author

The authors used fNIRS to measure the coherence (i.e. synchrony) in brain activity between familiar dyads watching a cartoon clip together, having a conversation, then watching another cartoon clip together. They then tested if (1) coherence was stronger between familiar dyads watching the clips together (i.e. co-experiencing) than randomly shuffled pairs, and (2) coherence between familiar dyads was stronger after the conversation than before the conversation. The study is well-motivated, and the manuscript is exceedingly well written. In particular, I really appreciated the comprehensive and well-organized literature on past work that the study builds on, as well as the detailed description of the methods.

Sara De Felice
Research Associate
Department of Psychology
Downing Place, CB2 3EB
Cambridge, UK

The main weaknesses of the paper are that the results do not survive correction for multiple comparisons, and that the analyses examine the effects of co-experience seem confounded by the effects of the conversation. I hope that the authors will find my comments below helpful:

1. The three results reported in the paper (increased brain synchrony in left TPJ and decreased brain synchrony in left SPL during co-experience; increased brain synchrony in right TPJ during co-experiencing after conversation) do not survive correction for multiple comparisons (uncorrected p = 0.04, 0.04, 0.03). Given 8 ROIs x 3 frequency bands = 24 tests, an uncorrected alpha threshold of 0.05 is rather liberal.

To be clear, I feel that journals should be open to publishing well-designed studies even if the results are not statistically significant or fail to survive for correction for multiple comparisons (see Scheel, Schijen & Lakens, 2023, Advances in Methods and Practices in Psychological Science). However, it is important to make this clear to readers upfront so that they are aware of the strength of the evidence supporting this claim. In the current manuscript, this is only mentioned in the second last paragraph of the discussion. I think it would be important to mention this in the abstract and the results section as well.

Yes, we agree with the reviewer and we have now been more explicit about the strength of our results, in the abstract (new text in bold):

*Findings reveal that real pairs showed increased brain synchrony over **right Dorso-Lateral Pre-Frontal cortex (DLPFC) and right Superior Parietal Lobe (SPL),** compared to pseudo pairs (who had never seen each other and watched the same movie at different times; **uncorrected for multiple comparisons).** In addition, **co-watching after a conversation was associated with greater synchrony over right TPJ compared to co-watching before a conversation**, and this effect was significantly higher in real pairs (who engaged in conversation with each other) compared to pseudo pairs (who had a conversation with someone else; **uncorrected for multiple comparisons).***

And in the result section (new text in bold):

*Full results are reported in Table 2 and 3 for all ROI and all frequency bands. Main findings are presented in Figure 5 and 6. **None of these results survived correction for multiple comparisons.***

UNIVERSITY OF CAMBRIDGE

Sara De Felice
Research Associate
Department of Psychology
Downing Place, CB2 3EB
Cambridge, UK

2. To examine if co-experience influenced synchrony, the authors compared mean brain coherence during co-watching phase 1 (i.e. before conversation) + co-watching phase 2 (after conversation) between the real and pseudo-dyads. Coherence in co-watching phase 2 would be influenced by both co-experience and the conversation. A cleaner test of the effects of co-experience would be to examine coherence in co-watching phase 1 only.

We thank the reviewer for pointing this out and suggesting a possible solution. We initially computed the mean of IBS during co-watching phases (co-watching of video 1 and video 2) for both real and pseudo (randomly shuffled) dyads, and conducted permutation testing to see whether there was any difference between real dyads watching a movie together versus pseudo dyads watching a movie with some random person. Our reasoning was that by averaging both videos, our signal would have been more robust to noise. However, as the reviewer pointed out, the mean across videos would also include some effects from conversation (as video 2 always happened post-conversation). We have therefore decided to replicate our previous analysis comparing real and pseudo dyads only for video 1. This way we got rid of the potential confounding factor of conversation. Results from this new analysis reveal significantly more IBS in real dyads over right DLPFC and right SPL (uncorrected for multiple comparisons). For transparency, we have included results from the previous analysis in Supplementary Materials., and indicated this in our results section (new text in bold):

A preliminary analysis used the mean of brain coherence across co-watching phases (i.e. pre- and post-conversation). This however may have been confounded by conversation-related effect. For transparency, results from this previous analysis are reported in supplementary materials.

We include ‘new’ results and discussion in the main manuscript (new text in bold). We have also updated Table 2 and updated Figure 5 to include new results (highlighted in yellow in the main manuscript document). Results section (new text in bold):

Brain coherence during co-watching of video 1 (phase 1) was significantly higher in real dyads compared to pseudo dyads over right DLPFC ($t = .11$; $p = .04$) and right SPL for the low frequency band ($t = .10$; $p = .03$). In other words, being physically in the same room during co-watching (real dyads) was associated with more brain coherence over the DLPFC and SPL in the right hemisphere, compared to what would be expected on average for processing the movie (pseudo dyads). No difference was found in brain coherence (in any frequency bands) for all other ROIs between real and pseudo dyads.

Sara De Felice
Research Associate
Department of Psychology
Downing Place, CB2 3EB
Cambridge, UK

Please note that to answer whether brain coherence change as a function of co-watching, we had initially conducted an analysis using the mean of brain coherence across co-watching phases (i.e. pre- and post-conversation). Results from this preliminary analysis are reported in supplementary materials.

Discussion section (new text in bold):

First, when comparing brain-to-brain synchrony between real dyads and pseudo dyads during the first co-watching phase, we found that real dyads showed greater synchrony over ***DLPFC and SPL in the right hemisphere. Increased neural synchrony over DLPFC during co-watching is consistent with previous studies showing that familiar pairs of people attending to stimuli together showed increased synchrony compared to both unfamiliar (shuffled) pairs and solo-experience (Azhari et al., 2020, 2021, 2022; Durnford et al., 2022). DLPFC has been found to play a crucial role in social bonding (Balconi et al., 2020), emotional resonance between people sharing painful experiences (Peng et al., 2021) and in regulating in-group dynamics (Yang et al., 2020). It is possible that our pairs of participants, being both familiar to each other and sharing the same experience of watching a movie together, engaged in some co-regulation which resulted in increased synchrony over this region. Similarly, sharing the same physical environment may have increased synchrony of their proprioceptive systems, resulting in greater synchrony over the right SPL (Ishida & Murata, 2007; Rizzolatti & Sinigaglia, 2010), compared to pseudo dyads who did not share the same spatio-temporal environment. Frontal-parietal circuit has also been found to play a key role in constructing mental models of self- and other-representations (Balconi et al., 2018; Dumas et al., 2010; Konvalinka et al., 2014), which may explain why these regions showed greater synchrony between people who were physically sharing an experience together.***

3. "This gave the spectrogram for each dyad in time-frequency space. Given the large range of frequencies, we selected three frequency bands of interest, namely high (0.1-0.2 Hz, i.e. 5-10 sec period), medium (0.03-0.1 Hz, i.e. 10-30 sec period), and low (0.02-0.03 Hz, i.e. 30-60 sec period; Figure 2C and 2D). This decision was informed by both a general agreement in the literature that different frequencies in hemodynamic rhythms are reflecting different cognitive processes (Cannon et al., 2014; Ward, 2003), and more specifically previous studies looking at brain coherence in social interaction contexts (Cui et al., 2011)."

Sara De Felice
Research Associate
Department of Psychology
Downing Place, CB2 3EB
Cambridge, UK

UNIVERSITY OF CAMBRIDGE

I found the motivation for examining the three frequency bands somewhat vague. Could the authors provide more background on what physiological and/or cognitive processes that might be tracked by each frequency band?

We thank the Reviewer for the comment. We have now included a more detailed explanations of our rationale for choosing those frequency bands in our methods section (new text in bold):

Based on a systematic investigation of wavelet coherence transform for fNIRS data, we have focused on frequency ranges that reflect fluctuations of hemodynamic activity (Zhang et al., 2020). Frequencies below 0.01 Hz are generally linked to noise, such as instrumental noise or vascular endothelial regulations (Pinti et al., 2019; Yücel et al., 2016) and therefore were not included in the analysis. Similarly, frequencies above 0.2 Hz are too fast for hemodynamic-related activity (Pinti et al., 2019). In fact, the hemodynamic response is 'slow', peaking roughly 5 seconds after neural activity.

We have examined inter-brain coherence at different time scales and in doing so we have considered ranges rather than single frequencies to account for between-subjects frequency variations (Zhao et al., 2023). Previous works have shown that interpersonal synchrony is spread across multiple frequencies (Nozawa et al., 2016) and that frequency components in the range 0.01-0.1 Hz reliably reflect components of neuronal origin (Zuo et al., 2010). Specifically, *we selected three frequency bands of interest, namely high (0.1-0.2 Hz, i.e. 5-10 sec period), medium (0.03-0.1 Hz, i.e. 10-30 sec period), and low (0.02-0.03 Hz, i.e. 30-60 sec period; Figure 2C and 2D). This decision was informed by both a general agreement in the literature that different frequencies in hemodynamic rhythms are reflecting different cognitive processes (Cannon et al., 2014; Ward, 2003), and more specifically previous studies looking at brain coherence in social interaction contexts (Cui et al., 2012).*

The high frequency range above 0.1 Hz was chosen to investigate if any synchronization occurred at higher frequencies. We believe that this would be plausible as previous fMRI studies suggested that there can still be neuronal contributions to the hemodynamic/BOLD signal above 0.1 Hz (Chen & Glover, 2015) and that these can reveal new properties of the brain organization in response to external inputs (Baria et al., 2011). Interpersonal synchronization above 0.1 Hz has also been reported in other hyperscanning studies (Nozawa et al., 2016).

Sara De Felice
Research Associate
Department of Psychology
Downing Place, CB2 3EB
Cambridge, UK

The medium frequency band was chosen to include the time scale of typical hemodynamic responses to a single event, therefore highlighting the synchronization of transient neural activation across the two brains (Sasai et al., 2011).

The low frequency band was chosen to investigate if any synchronization between spontaneous neuronal activities is observed between participants and also between more sustained hemodynamic activity within our long task cycle (2 minutes).
