# Supplementary material for: Having a chat and then watching a movie: how social interaction synchronises our brains during co-watching
Source: Oxf Open Neurosci. 2024 Mar 25;3:kvae006. doi: 10.1093/oons/kvae006 (PMC11069416; doi:10.1093/oons/kvae006)
Supplement: Web_Material_kvae006 [file web_material_kvae006.zip › Reviewers comments - Supplementary Material - OXFNSC-2023-009 .docx]

**REVIEWER COMMENTS**

**OXFNSC-2023-009 - Social interaction increases brain synchrony during co-watching of novel movies**

**Reviewer 1**

Comments to the Author

This paper is clearly written and investigates an interesting question: whether mere social interactions (unrelated conversations), is required to increase the brain-to-brain synchrony between pairs of participants when they watch movies before and after the conversations. The fNIRS results showed different levels of neural synchrony between real dyads and pseudo-dyads, as well as increased synchrony between real dyads before and after conversations.

I enjoyed reading this paper overall, but I have a couple major concerns:

- The authors seem to be missing a large body of literature in their review.

+ For example, the authors mentioned that “the *only* attempt to answer the question of whether social interaction modulates ISC is an fMRI study by (Sievers et al., n.d.)”. I think this statement is seriously oversimplifying the research in this field, since there are many studies looking at how social interactions affect brain synchrony. A few examples:

- Yun et al. (2012, https://doi.org/10.1038/srep00959): This study demonstrated that pairs of participants showed increased brain synchrony (measured by EEG) after a cooperative task, which involved social interaction without conversations. I believe this study answered the authors’ research question in the current paper to an extent.

- Spiegelhalder et al. (2014, https://doi.org/10.1016/j.bbr.2013.10.015) showed increased brain synchrony (fMRI) during verbal interactions.

- Pan et al. (2023, https://doi.org/10.1093/cercor/bhac127) showed triads of participants had increased neural synchronization (fNIRS) after cooperations.

- Kelson et al. (2022, https://doi.org/10.1016/j.neubiorev.2020.09.008) provided a review on EEG/fNIRS hyperscanning studies of verbal communications.

- Tsoi et al. (2022, https://doi.org/10.1111/spc3.12707) also provided a relevant review on some fMRI studies related to this topic.

+ Considering this body of literature, the novel insights provided by the current study seems to be somewhat limited, even though the results of this study are still quite interesting. It might be necessary for the authors to substantially reframe their introduction/literature review part to cover the past research on this topic more comprehensively, and to evaluate their study’s contribution accurately.

- This study compared dyads who were familiar with each other (friends, flatmates or partners) who co-watched movies and had conversations, to pseudo-dyads who were not familiar with each other who did not co-watch or have conversations. It remains quite unclear whether the results arise from pairs being familiar with each other (as found in Parkinson et al., 2018), or them co-watching movies, or them having conversations, or all three. The authors talked about this in their discussions, but much of the introduction and results was still framed such that it seems like the familiarity was not a factor.

+ Ideally, another experiment could be performed with unfamiliar dyads going through the same procedure, so that their results could be compared. I think this would make the paper a lot stronger and much more interesting. Even better, there could be yet another experiment where participants who only co-watched movies while not engaging in any conversations, so that the three factors could be separated.

+ Alternatively, I think the authors may also try to (substantially) reframe the paper so that the focus is not on comparing between real and pseudo-dyads, but on comparing just the real dyads between their co-watching phase 1 versus phase 2 (i.e., before versus after having a conversation).

A few other minor points:

- The authors mentioned that a separator was placed in between the two participants while they were co-watching. It seems like this would make the situation somewhat unnatural. How would placing a separator empirically different from placing two participants in separate cubicles? Or neighboring rooms? To what extent would this still count as a “co-watching” experience? I wonder if the authors could justify this in more details (if the only purpose was to prevent social interactions, is it not sufficient to simply tell them to stay still and not talk?).

- It would be nice if the authors can elaborate on the participants’ conversations and how they made sure it was unrelated to the cartoon. How was it “semi-structured”? Did the experimenter set a topic for the participants?

- The authors may want to check their reference list. For example, Cui et al. seems to be published in 2012, but when the authors cited this paper they mostly wrote 2011 except for one occasion. Also Sievers et al. has been out as a preprint since 2020, and the authors should cite it accordingly (rather than using ”n.d.”)

- There is a typo in the last few lines of page 5 (“than”?).

**Reviewer 2**

Comments to the Author

The authors used fNIRS to measure the coherence (i.e. synchrony) in brain activity between familiar dyads watching a cartoon clip together, having a conversation, then watching another cartoon clip together. They then tested if (1) coherence was stronger between familiar dyads watching the clips together (i.e. co-experiencing) than randomly shuffled pairs, and (2) coherence between familiar dyads was stronger after the conversation than before the conversation. The study is well-motivated, and the manuscript is exceedingly well written. In particular, I really appreciated the comprehensive and well-organized literature on past work that the study builds on, as well as the detailed description of the methods.

The main weaknesses of the paper are that the results do not survive correction for multiple comparisons, and that the analyses examine the effects of co-experience seem confounded by the effects of the conversation. I hope that the authors will find my comments below helpful:

1. The three results reported in the paper (increased brain synchrony in left TPJ and decreased brain synchrony in left SPL during co-experience; increased brain synchrony in right TPJ during co-experiencing after conversation) do not survive correction for multiple comparisons (uncorrected p = 0.04, 0.04, 0.03). Given 8 ROIs x 3 frequency bands = 24 tests, an uncorrected alpha threshold of 0.05 is rather liberal.

To be clear, I feel that journals should be open to publishing well-designed studies even if the results are not statistically significant or fail to survive for correction for multiple comparisons (see Scheel, Schijen & Lakens, 2023, Advances in Methods and Practices in Psychological Science). However, it is important to make this clear to readers upfront so that they are aware of the strength of the evidence supporting this claim. In the current manuscript, this is only mentioned in the second last paragraph of the discussion. I think it would be important to mention this in the abstract and the results section as well.

2. To examine if co-experience influenced synchrony, the authors compared mean brain coherence during co-watching phase 1 (i.e. before conversation) + co-watching phase 2 (after conversation) between the real and pseudo-dyads. Coherence in co-watching phase 2 would be influenced by both co-experience and the conversation. A cleaner test of the effects of co-experience would be to examine coherence in co-wathcing phase 1 only.

3. “This gave the spectrogram for each dyad in time-frequency space. Given the large range of frequencies, we selected three frequency bands of interest, namely high (0.1-0.2 Hz, i.e. 5-10 sec period), medium (0.03-0.1 Hz, i.e. 10-30 sec period), and low (0.02-0.03 Hz, i.e. 30-60 sec period; Figure 2C and 2D). This decision was informed by both a general agreement in the literature that different frequencies in hemodynamic rhythms are reflecting different cognitive processes (Cannon et al., 2014; Ward, 2003), and more specifically previous studies looking at brain coherence in social interaction contexts (Cui et al., 2011).”

I found the motivation for examining the three frequency bands somewhat vague. Could the authors provide more background on what physiological and/or cognitive processes that might be tracked by each frequency band?

**Decision letter**

03-Dec-2023

Dear Dr. De Felice,

Manuscript ID OXFNSC-2023-009 entitled "Social interaction increases brain synchrony during co-watching of novel movies" which you submitted to the Oxford Open Neuroscience, has been reviewed. The comments of the reviewer(s) are included at the bottom of this letter.

Please note that this journal operates with transparent peer review. This means that if your submission is eventually accepted for publication, the full peer review history of your article will publish online alongside your article. This includes reviewer comments, editor decision letters, and your author responses.

The reviewers request major revisions to your manuscript that include placing the present study within the context of a broader literature and the appropriate use of statistical tests that include corrections for number of comparisons. I invite you to respond to the reviewer(s)' comments and revise your manuscript within the next 60 days from today date.

To revise your manuscript, log into https://mc.manuscriptcentral.com/oxfnsc and enter your Author Centre, where you will find your manuscript title listed under "Manuscripts with Decisions." Under "Actions," click on "Create a Revision." Your manuscript number has been appended to denote a revision.

You may also click the below link to start the revision process (or continue the process if you have already started your revision) for your manuscript. If you use the below link you will not be required to login to ScholarOne Manuscripts.

*** PLEASE NOTE: This is a two-step process. After clicking on the link, you will be directed to a webpage to confirm. ***

https://mc.manuscriptcentral.com/oxfnsc?URL_MASK=f5158d08bd99406aa0afdd1c01f523b7

You will be unable to make your revisions on the originally submitted version of the manuscript. Instead, revise your manuscript using a word processing program and save it on your computer. Please also highlight the changes to your manuscript within the document by using the track changes mode in MS Word or by using bold or colored text.

Once the revised manuscript is prepared, you can upload it and submit it through your Author Centre.

When submitting your revised manuscript, you will be able to respond to the comments made by the reviewer(s) in the space provided. You can use this space to document any changes you make to the original manuscript. In order to expedite the processing of the revised manuscript, please be as specific as possible in your response to the reviewer(s).

IMPORTANT: Your original files are available to you when you upload your revised manuscript. Please delete any redundant files before completing the submission.

Because we are trying to facilitate timely publication of manuscripts submitted to the Oxford Open Neuroscience, your revised manuscript should be uploaded within the next 60 days from today date. If it is not possible for you to submit your revision in a reasonable amount of time, we may have to consider your paper as a new submission.

Once again, thank you for submitting your manuscript to the Oxford Open Neuroscience and I look forward to receiving your revision.

Sincerely,

Dr. Alicia Izquierdo

Senior Editor, Oxford Open Neuroscience

aizquie@psych.ucla.edu

Reviewer: 1

Comments to the Author

This paper is clearly written and investigates an interesting question: whether mere social interaction (unrelated conversations), is required to increase the brain-to-brain synchrony between pairs of participants when they watch movies before and after the conversations. The fNIRS results showed different levels of neural synchrony between real dyads and pseudo-dyads, as well as increased synchrony between real dyads before and after conversations.

I enjoyed reading this paper overall, but I have a couple major concerns:

- The authors seem to be missing a large body of literature in their review.

+ For example, the authors mentioned that “the *only* attempt to answer the question of whether social interaction modulates ISC is an fMRI study by (Sievers et al., n.d.)”. I think this statement is seriously oversimplifying the research in this field, since there are many studies looking at how social interactions affect brain synchrony. A few examples:

- Yun et al. (2012, https://doi.org/10.1038/srep00959): This study demonstrated that pairs of participants showed increased brain synchrony (measured by EEG) after a cooperative task, which involved social interaction without conversations. I believe this study answered the authors’ research question in the current paper to an extent.

- Spiegelhalder et al. (2014, https://doi.org/10.1016/j.bbr.2013.10.015) showed increased brain synchrony (fMRI) during verbal interactions.

- Pan et al. (2023, https://doi.org/10.1093/cercor/bhac127) showed triads of participants had increased neural synchronization (fNIRS) after cooperations.

- Kelson et al. (2022, https://doi.org/10.1016/j.neubiorev.2020.09.008) provided a review on EEG/fNIRS hyperscanning studies of verbal communications.

- Tsoi et al. (2022, https://doi.org/10.1111/spc3.12707) also provided a relevant review on some fMRI studies related to this topic.

+ Considering this body of literature, the novel insights provided by the current study seems to be somewhat limited, even though the results of this study are still quite interesting. It might be necessary for the authors to substantially reframe their introduction/literature review part to cover the past research on this topic more comprehensively, and to evaluate their study’s contribution accurately.

- This study compared dyads who were familiar with each other (friends, flatmates or partners) who co-watched movies and had conversations, to pseudo-dyads who were not familiar with each other who did not co-watch or have conversations. It remains quite unclear whether the results arise from pairs being familiar with each other (as found in Parkinson et al., 2018), or them co-watching movies, or them having conversations, or all three. The authors talked about this in their discussions, but much of the introduction and results was still framed such that it seems like the familiarity was not a factor.

+ Ideally, another experiment could be performed with unfamiliar dyads going through the same procedure, so that their results could be compared. I think this would make the paper a lot stronger and much more interesting. Even better, there could be yet another experiment where participants who only co-watched movies while not engaging in any conversations, so that the three factors could be separated.

+ Alternatively, I think the authors may also try to (substantially) reframe the paper so that the focus is not on comparing between real and pseudo-dyads, but on comparing just the real dyads between their co-watching phase 1 versus phase 2 (i.e., before versus after having a conversation).

A few other minor points:

- The authors mentioned that a separator was placed in between the two participants while they were co-watching. It seems like this would make the situation somewhat unnatural. How would placing a separator empirically different from placing two participants in separate cubicles? Or neighboring rooms? To what extent would this still count as a “co-watching” experience? I wonder if the authors could justify this in more details (if the only purpose was to prevent social interactions, is it not sufficient to simply tell them to stay still and not talk?).

- It would be nice if the authors can elaborate on the participants’ conversations and how they made sure it was unrelated to the cartoon. How was it “semi-structured”? Did the experimenter set a topic for the participants?

- The authors may want to check their reference list. For example, Cui et al. seems to be published in 2012, but when the authors cited this paper they mostly wrote 2011 except for one occasion. Also Sievers et al. has been out as a preprint since 2020, and the authors should cite it accordingly (rather than using ”n.d.”)

- There is a typo in the last few lines of page 5 (“than”?).

Reviewer: 2

Comments to the Author

The authors used fNIRS to measure the coherence (i.e. synchrony) in brain activity between familiar dyads watching a cartoon clip together, having a conversation, then watching another cartoon clip together. They then tested if (1) coherence was stronger between familiar dyads watching the clips together (i.e. co-experiencing) than randomly shuffled pairs, and (2) coherence between familiar dyads was stronger after the conversation than before the conversation. The study is well-motivated, and the manuscript is exceedingly well written. In particular, I really appreciated the comprehensive and well-organized literature on past work that the study builds on, as well as the detailed description of the methods.

The main weaknesses of the paper are that the results do not survive correction for multiple comparisons, and that the analyses examine the effects of co-experience seem confounded by the effects of the conversation. I hope that the authors will find my comments below helpful:

1. The three results reported in the paper (increased brain synchrony in left TPJ and decreased brain synchrony in left SPL during co-experience; increased brain synchrony in right TPJ during co-experiencing after conversation) do not survive correction for multiple comparisons (uncorrected p = 0.04, 0.04, 0.03). Given 8 ROIs x 3 frequency bands = 24 tests, an uncorrected alpha threshold of 0.05 is rather liberal.

To be clear, I feel that journals should be open to publishing well-designed studies even if the results are not statistically significant or fail to survive for correction for multiple comparisons (see Scheel, Schijen & Lakens, 2023, Advances in Methods and Practices in Psychological Science). However, it is important to make this clear to readers upfront so that they are aware of the strength of the evidence supporting this claim. In the current manuscript, this is only mentioned in the second last paragraph of the discussion. I think it would be important to mention this in the abstract and the results section as well.

2. To examine if co-experience influenced synchrony, the authors compared mean brain coherence during co-watching phase 1 (i.e. before conversation) + co-watching phase 2 (after conversation) between the real and pseudo-dyads. Coherence in co-watching phase 2 would be influenced by both co-experience and the conversation. A cleaner test of the effects of co-experience would be to examine coherence in co-watching phase 1 only.

3. “This gave the spectrogram for each dyad in time-frequency space. Given the large range of frequencies, we selected three frequency bands of interest, namely high (0.1-0.2 Hz, i.e. 5-10 sec period), medium (0.03-0.1 Hz, i.e. 10-30 sec period), and low (0.02-0.03 Hz, i.e. 30-60 sec period; Figure 2C and 2D). This decision was informed by both a general agreement in the literature that different frequencies in hemodynamic rhythms are reflecting different cognitive processes (Cannon et al., 2014; Ward, 2003), and more specifically previous studies looking at brain coherence in social interaction contexts (Cui et al., 2011).”

I found the motivation for examining the three frequency bands somewhat vague. Could the authors provide more background on what physiological and/or cognitive processes that might be tracked by each frequency band?

**Author response**

Dear Dr. Izquierdo,

thank you for your response and for considering our manuscript for submission in Oxford Open Neuroscience Journal.

We believe we have addressed all the reviewers' comments. Please refer to the uploaded file 'Cover letter & response to reviewers' for a detailed response to each comment.

**OXFNSC-2023-009.R1 - Having a chat and then watching a movie: how social interaction synchronises our brains during co-watching**

**Reviewer 1**

Comment to the Author

I appreciate the detailed response from the authors. I think the paper has improved significantly, and my concerns have been fully addressed.

**Reviewer 2**

Comments to the Author

I am satisfied with the revisions. Please note that Fig. 1S did not seem to render/convert to PDF properly.

**Decision letter**

07-Mar-2024

Dear Dr. De Felice,

It is a pleasure to accept your revised manuscript entitled "Having a chat and then watching a movie: how social interaction synchronises our brains during co-watching" in its current form for publication in the Oxford Open Neuroscience. The comments of the reviewers who reviewed your manuscript are included at the foot of this letter. In particular, please upload a clean copy (with no tracked changes) and ensure that all Figures are of sufficient quality for the final version sent to print.

Please note that this journal operates with transparent peer review. This means that the full peer review history of your article will publish online alongside your article. This includes reviewer comments, editor decision letters, and your author responses.

Next steps

You will receive an email from no-reply@scipris.com within roughly one week. This is your invitation to sign up for an account with SciPris, Oxford University Press’ author portal hosted by Aptara. You will need to create an account if you do not already hold one. Please register or log into your account and follow the online instructions which will guide you through signing your licence and paying the APC. The email and the portal have clearly signposted support options if you need any help during this process.

Please note that SciPris is a completely different system from ScholarOne, so your credentials to submit your manuscript here will not work there. Once you’ve created a SciPris account, you will be able to use it whenever you publish with Oxford Open Neuroscience or any OUP journal. Please note that OUP will only ever request payment for applicable fees be made via SciPris or to an OUP bank account. If you ever have concerns about the legitimacy of a request, please do not hesitate to contact a customer services agent via the SciPris portal or directly via oupsupport@scipris.com.

Thank you for your excellent contribution. On behalf of the Editors of the Oxford Open Neuroscience, we look forward to your continued contributions to the Journal.

Sincerely,

Dr. Alicia Izquierdo

Senior Editor, Oxford Open Neuroscience

aizquie@psych.ucla.edu

Reviewer: 2

Comments to the Author

I am satisfied with the revisions. Please note that Fig. 1S did not seem to render/convert to PDF properly.

Reviewer: 1

Comments to the Author

I appreciate the detailed response from the authors. I think the paper has improved significantly, and my concerns have been fully addressed.
